# Plasma exosome microRNAs are indicative of breast cancer

**DOI:** 10.1186/s13058-016-0753-x

**Published:** 2016-09-08

**Authors:** Bethany N. Hannafon, Yvonne D. Trigoso, Cameron L. Calloway, Y. Daniel Zhao, David H. Lum, Alana L. Welm, Zhizhuang J. Zhao, Kenneth E. Blick, William C. Dooley, W. Q. Ding

**Affiliations:** 1 Department of Pathology, University of Oklahoma Health Sciences Center, Oklahoma City, OK 73104 USA; 2 Department of Biostatistics and Epidemiology, University of Oklahoma Health Sciences Center, Oklahoma City, OK 73104 USA; 3 Oklahoma Medical Research Foundation, Oklahoma City, OK 73104 USA; 4 Huntsman Cancer Institute, University of Utah, Salt Lake City, UT 84112 USA; 5 Department of Surgery, University of Oklahoma Health Sciences Center, Oklahoma City, OK 73104 USA; 6 Peggy and Charles Stephenson Cancer Center, Oklahoma City, OK 73104 USA

**Keywords:** Exosomes, Breast cancer, microRNA, Biomarker, Patient-derived xenograft

## Abstract

**Background:**

microRNAs are promising candidate breast cancer biomarkers due to their cancer-specific expression profiles. However, efforts to develop circulating breast cancer biomarkers are challenged by the heterogeneity of microRNAs in the blood. To overcome this challenge, we aimed to develop a molecular profile of microRNAs specifically secreted from breast cancer cells. Our first step towards this direction relates to capturing and analyzing the contents of exosomes, which are small secretory vesicles that selectively encapsulate microRNAs indicative of their cell of origin. To our knowledge, circulating exosome microRNAs have not been well-evaluated as biomarkers for breast cancer diagnosis or monitoring.

**Methods:**

Exosomes were collected from the conditioned media of human breast cancer cell lines, mouse plasma of patient-derived orthotopic xenograft models (PDX), and human plasma samples. Exosomes were verified by electron microscopy, nanoparticle tracking analysis, and western blot. Cellular and exosome microRNAs from breast cancer cell lines were profiled by next-generation small RNA sequencing. Plasma exosome microRNA expression was analyzed by qRT-PCR analysis.

**Results:**

Small RNA sequencing and qRT-PCR analysis showed that several microRNAs are selectively encapsulated or highly enriched in breast cancer exosomes. Importantly, the selectively enriched exosome microRNA, human miR-1246, was detected at significantly higher levels in exosomes isolated from PDX mouse plasma, indicating that tumor exosome microRNAs are released into the circulation and can serve as plasma biomarkers for breast cancer. This observation was extended to human plasma samples where miR-1246 and miR-21 were detected at significantly higher levels in the plasma exosomes of 16 patients with breast cancer as compared to the plasma exosomes of healthy control subjects. Receiver operating characteristic curve analysis indicated that the combination of plasma exosome miR-1246 and miR-21 is a better indicator of breast cancer than their individual levels.

**Conclusions:**

Our results demonstrate that certain microRNA species, such as miR-21 and miR-1246, are selectively enriched in human breast cancer exosomes and significantly elevated in the plasma of patients with breast cancer. These findings indicate a potential new strategy to selectively analyze plasma breast cancer microRNAs indicative of the presence of breast cancer.

**Electronic supplementary material:**

The online version of this article (doi:10.1186/s13058-016-0753-x) contains supplementary material, which is available to authorized users.

## Background

Breast cancer is the most diagnosed non-skin cancer and the second leading cause of cancer death in women in the USA [1]. The 5-year survival rate for women with early-stage breast cancer is 99 %, which is significantly higher than in those with advanced-stage invasive breast cancer (IBC) (24 % among patients with distant metastasis) [1], thus early detection is still a key to improving breast cancer outcomes.

Despite significant progress in breast cancer research, reliable biomarkers have yet to be identified. The current methods for early breast cancer detection, namely clinical examination and mammography, have certain limitations in their sensitivity and specificity. For example, mammography can detect only 70–90 % of breast lesions [2], with a false-positive rate of up to 31 % [3]. microRNAs are promising candidate biomarkers due to their cancer-specific expression profiles and roles in cancer initiation and progression. Common microRNA expression changes are observed in breast cancer tissues at both the pre-invasive and invasive stage [4, 5] and in serum from patients with advanced breast cancer [6]. Circulating microRNAs that are aberrantly expressed in tissues and serum/plasma have been explored for the purpose of developing biomarkers for breast cancer [7–9]. However, this has not been successful, potentially due to the inherent heterogeneity of the microRNA populations in the blood. New strategies in selective detection of circulating microRNAs are necessary to further the development of microRNAs as biomarkers for breast cancer.

Exosomes are endosome-derived nanometer-sized (50–150 nm) vesicles that are secreted from many cell types and contain cellular proteins, lipids and microRNAs [10]. Cancer cells secrete exosomes [11, 12], and the transfer of exosomes from primary tumors to the circulation has been demonstrated in various model systems [11, 13]. Furthermore, certain microRNA species contained in exosomes from cultured breast cancer cells or ductal lavage samples differ from those secreted from normal mammary epithelial cells [14], suggesting the potential use of these exosome microRNAs as biomarkers for the detection of malignant breast disease. However, to our knowledge, circulating exosome microRNAs have not been well-evaluated as biomarkers for breast cancer diagnosis or monitoring. In this study, we characterized the microRNA content in exosomes derived from breast cancer cells, and evaluated certain exosome microRNA species in plasma exosome samples from patient-derived xenograft (PDX) mice and patients with breast cancer. The findings from our study support the notion that plasma exosome microRNAs could serve as biomarkers for the presence of human breast cancer.

## Methods

### Cell culture

The human mammary epithelial cell line MCF10A, and breast cancer cell lines MCF7, ZR-75-1, T47D , BT20, BT-474, SK-BR-3, and MDA-MB-231 were obtained from the American Type Culture Collection (Manassas, VA, USA). MCF10A cells were cultured in DMEM/F12 supplemented with 5 % exosome-depleted horse serum, 20 ng/ml epithelial growth factor, 0.5 mg/ml hydrocortisone, 100 ng/ml cholera toxin, 10 μg/ml insulin, 100 IU/ml penicillin, and 100 μg/ml streptomycin. MCF7, ZR-75-1, BT20 and MDA-MB-231 cells were cultivated in DMEM supplemented with 10 % exosome-depleted fetal bovine serum (FBS), 100 IU/ml penicillin and 100 μg/ml streptomycin (Corning/Mediatech, Inc. Manassas, VA, USA). BT-474 and T47D cells were cultivated in RPMI medium supplemented with 10 % exosome-depleted FBS, 100 IU/ml penicillin and 100 μg/ml streptomycin. SK-BR-3 cells were cultivated in McCoy’s 5A medium supplemented with 10 % exosome-depleted FBS, 100 IU/ml penicillin and 100 μg/ml streptomycin. Exosome-depleted FBS and horse serum were prepared by pelleting the serum exosomes by ultracentrifugation at 100,000 × *g* for 2 h at 4 °C, and the resulting supernatant was filtered through a 0.2-μm pore filter. Cells were routinely maintained in a humidified chamber at 37 °C and 5 % CO_2_.

### Patient-derived orthotopic xenograft plasma collection

PDX mice were produced and maintained as previously described [15]. For terminal plasma collection the mice were euthanized by CO_2_ asphyxiation and were placed on their back (dorsal recumbence). The chest was wetted with 70 % ethanol and the thoracic cavity was exposed by an incision through the ribs. Blood was collected with a 29G insulin syringe (Exel Int., 26028) and dispensed into a microvette (EDTA) tube (SARSTEDT, Microvette 200K3E). Blood was mixed gently in the tube to ensure exposure to EDTA-coated walls. The plasma was separated by centrifuging the blood sample at 2000 rcf for 15 minutes at room temperature. The clear top layer was transferred to a labeled tube and stored at -80 °C.

### Patient plasma samples

Plasma samples were collected from women with no history of breast cancer (mean age = 42 years) and provided by the Oklahoma Blood Institute, Oklahoma City, OK, USA. Breast cancer plasma samples were collected from women who were seen at the Stephenson Cancer Center at the University of Oklahoma, Oklahoma City, OK and underwent primary tumor biopsy or resection. The University of Oklahoma Health Sciences Center Human Research Participant Protection Institutional Review Board approved the study (#4381) and written informed consent was obtained from all participants.

A total of 32 plasma samples was utilized: 16 healthy plasma and 16 breast cancer plasma samples. Blood was collected and dispensed into an EDTA collection tube and mixed gently to ensure exposure to the EDTA-coated walls. Plasma was separated by centrifugation on a standard clinical centrifuge at 2000 rcf for 15 minutes at room temperature. The clear top layer was transferred to a labeled tube, and stored at -80 °C. Clinicopathological factors and clinical stages were classified using the tumor, node, metastasis (TNM) system (AJCC 7^th^ edition). All data for the breast cancer samples, including age, tumor size, clinical stage, histological grade, hormone receptor, and human epidermal growth factor receptor 2 (HER2) amplification status, were obtained from the clinical and pathological records.

### Exosome isolation

Exosomes were isolated utilizing a combination of centrifugation, ultracentrifugation, and filtration as we have previously described [16], or with the Exoquick-TC reagent (System Biosciences, Mountain View, CA, USA) following the manufacturer’s protocol. For ultracentrifugation isolation, conditioned cell culture medium was collected and centrifuged at 10,000 × *g* for 30 minutes at 4 °C, to remove cells and large debris. The supernatant was filtered using a 0.22-μm pore filter and the exosomes were pelleted at 100,000 × *g* for 1 h at 4 °C. The exosome pellet was washed with 10 ml of 1 × PBS and pelleted again by centrifugation at 100,000 × *g* for 1 h at 4 °C. The resulting pellet was either suspended in 1 × PBS for whole exosome applications or further processed for RNA or protein extraction. Plasma exosomes were isolated using the Exoquick reagent (System Biosciences, Mountain View, CA, USA) following the manufacturer’s protocol. The resulting exosome pellet was suspended in PBS and exosome concentration was estimated by Bradford assay.

### Western blot analysis

Total exosome protein was prepared by re-suspending the exosomes in RIPA Buffer (50 mM Tris-HCl pH 7.4, 150 mM NaCl, 0.5 % sodium deoxycholate, 1 % NP-40, and 0.1 % sodium dodecyl sulfate) containing 1 mM phenlymethylsulfonyl fluoride, 5 μg/ml leupeptin, 2 μg/ml aprotinin, and 1 μg/ml pepstatin A. About 30–40 μg of protein from each sample was separated under non-reducing conditions on a 10 % SDS-PAGE gel, transferred to a polyvinylidene fluoride (PVDF) membrane, and blotted with an antibody against CD63 (sc-5275, Santa Cruz Biotechnology, Santa Cruz, CA, USA).

### Electron microscopy and immunogold labeling

Whole exosomes suspended in 1 × PBS were fixed in 2 % paraformaldehyde. The fixed sample was absorbed onto formvar-coated copper grids for 20 minutes in a dry environment. Samples were then fixed in 1 % glutaraldehyde for 5 minutes. After being rinsed in distilled water, samples were stained with uranyl oxalate for 5 minutes followed by methyl cellulose uranyl acetate for 10 minutes on ice. Excess liquid was wicked off the grid using filter paper, and grids were stored at room temperature until imaging. For immunogold labeling, exosomes were fixed in 2 % paraformaldehyde. Samples were absorbed onto formvar-coated copper grids for 20 minutes in a dry environment and washed with PBS three times. Samples then underwent four washes in 50 mM glycine followed by a 10-minute blocking step. Exosomes were incubated with CD63 (Santa Cruz Biotechnology, Santa Cruz, CA, USA) primary antibody for 30 minutes, and then samples were washed in washing buffer six times. Samples were incubated in secondary antibody conjugated to 10 nM gold particles for 20 minutes. Finally, samples were washed in PBS, stabilized with glutaraldehyde, washed in water, and counterstained with uranyl oxalate and methyl cellulose uranyl acetate. Imaging was performed using a Hitachi H7600 microscope.

### Nanoparticle tracking analysis

Isolated exosomes were diluted in PBS and analyzed using the Nanosight NS300 System (Malvern Instruments, UK) equipped with a blue laser (405 nm). Nanoparticles illuminated by the laser and their movement under Brownian motion was captured for 60 seconds. Videos were analyzed using the Nanosight Tracking Analysis (NTA) software to provide particle concentrations and size distribution profiles. Triplicate measurements were recorded for each sample. Size distribution and concentration profiles were averaged across replicates to derive the representative size distribution profiles.

### RNA extraction

Total RNA was extracted from exosome pellet using the TRIzol reagent (Invitrogen/Life Technologies) following the manufacturer’s protocol. RNA concentration was quantitated using the NanoDrop ND-100 Spectrophotometer (NanoDrop Technologies, Wilmington, DE, USA).

### Small RNA library preparation and next generation sequencing

Small RNA libraries were constructed using New England Biolabs (NEB) NEBNext Multiplex Small RNA Library Prep Set for Illumina sequencers and the NEB standard protocol. Individual libraries were constructed using 1 μg of total RNA isolated from each sample. Each library was indexed in order to multiplex four samples per sequencing run on the Illumina MiSeq platform using MiSeq 50 cycle Reagent Kits v2. A minimum of 17 million 50-bp sequencing reads were collected from each sample and data were analyzed using Genesifter software (formerly Geospiza) (PerkinElmer, Santa Clara, CA, USA). Raw data for each sample were aligned to the most recent mirBASE database (mirBase.org [17]) with remaining reads aligned to the most recent human genome (hg18 build [18]) build in order to identify previously unknown regions that may encode for unique miRNAs. Pairwise comparison of the alignment results was done using Genesifter for identification of miRNAs that are differentially expressed at a significant level, i.e., upregulated or downregulated.

### Quantitative real-time reverse transcription PCR

For microRNA expression analysis complementary DNA from 10 ng of total RNA was synthesized by the addition of a microRNA-specific 5X reverse transcription stem-loop primer and the TaqMan microRNA Reverse Transcription Kit, according to the manufacturer’s instructions. Real-Time PCR was performed by diluting the complementary cDNA product in 2X TaqMan Universal Master Mix II (with UNG) and 20X TaqMan microRNA Expression Assay for each mature microRNA to be measured: miR-21 (ID:000397), miR-122 (ID:002245), miR-451 (ID:001141) let-7a (ID:000377) and miR-1246 (custom assay ID:CSQJA23). All reagents and primers were from ThermoFisher/Life Technologies. The small ribonuclear RNA RNU6B (ID:001093) served as a microRNA expression normalization control for cellular microRNA expression analysis.

Because no internal controls for exosome microRNA analysis have been established, we used a synthetic *Caenorhabditis elegans* miR-54 (cel-miR-54) RNA oligonucleotide (Integrated DNA Technologies, Coralville, IA, USA) as a spike-in control. Cel-miR-54 has previously been shown not to affect human microRNA detection [19]. The cel-miR-54 (0.25 nM) oligonucleotide was spiked into each RNA sample prior to complementary DNA synthesis and Real-Time PCR was performed using the TaqMan microRNA assay (ID:001361, Life Technologies). PCR reactions were run in triplicate on the Bio-Rad CFX 96 Real-Time PCR (Bio-Rad, Hercules, CA, USA) instrument under the following conditions: hold at 95 °C for 10 minutes, then 40 cycles of 95 °C for 15 s and 60 °C for 1 minute. A standard curve of cel-miR-54 was generated by five-fold serial dilution of cDNA. Absolute expression values were determined by linear regression analysis. Copy number values were computed based on the equation:

microRNA copies = (ng cel-miR-54 × 6.022 × 10^23^)/(24 nt × (1 × 10^9^) × 650).

### Immunoaffinity magnetic bead-based exosome isolation

Dynabeads Protein G (#10003D, ThermoFisher, Waltham, MA, USA) were first prepared by binding 20 μg of CD63 primary antibody diluted in 1 × PBS with 0.02 % Tween-20 to 100 μl of Dynabeads. Bead-antibody complex was incubated with rotation for 10 minutes at room temperature. To remove unbound antibody, the tube was placed on a magnet and the supernatant was removed. The antibody-coated beads were suspended in 400 μl of 1 × PBS with 0.02 % Tween-20. Exosome samples were brought to a concentration of 50 μg in 200 μl in 1 × PBS with 0.1 % bovine serum albumin (isolation buffer). Antibody-complexed beads (200 μl) were transferred to a fresh 2.0-ml tube, washed with 500 μl of isolation buffer, placed on a magnet, and the supernatant was discarded. The tube was removed from the magnet and the diluted exosome samples were added to the tube containing the beads, mixed gently by pipetting, and incubated overnight (18–22 h) at 4 °C, with gentle rotation. After incubation, the tube was gently centrifuged to collect the sample, placed on the magnet, and the supernatant was removed and discarded. Bead-bound exosomes were washed twice by adding 300–400 μl of isolation buffer and mixed gently by pipetting; the tube was then applied to the magnet for 1 minute and the supernatant was discarded. RNA was extracted from the exosome-bound beads following the manufacturer’s protocol by directly applying TRIzol reagent to the beads after the second wash.

### Statistical analysis

Statistical analyses were completed using GraphPad Prism software (GraphPad Software, Inc. La Jolla, CA, USA). When appropriate Student’s *t* test was used to determine statistically significant differences among control and experimental groups, with a *p* value <0.05 as the level of significance. For each patient sample and each microRNA analyzed, the average of three replicate expression values was computed. Receiver operating characteristic (ROC) curves were constructed using each microRNA expression value individually or jointly. The area under the curve (AUC) with 95 % CI was calculated for each ROC curve. The Wilcoxon-Mann-Whitney test was used to test the null hypothesis that the AUC is equal to 0.5 (i.e., no predictive power).

## Results

### microRNAs are selectively enriched in exosomes secreted from breast cancer cells

**Fig. 1:**
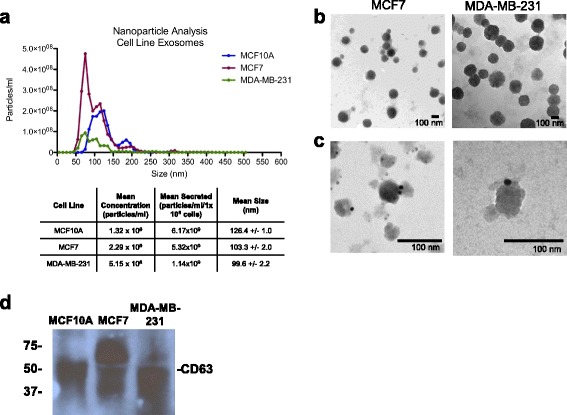
Characterization of exosomes from breast cancer cell lines. Exosomes were isolated from the conditioned media of MCF10A, MCF7, and MDA-MB-231 cells. **a** Nanoparticle tracking analysis of 50X diluted MCF10A, MCF7 and MDA-MB-231 exosomes. **b** MCF7 and MDA-MB-231 exosomes were visualized by electron microscopy (×30,000). **c** MCF7 and MDA-MB-231 exosomes were immunogold-labeled and visualized by electron microscopy of human CD63-gold particles (10 nM gold) (*right* × 40,000; *left* × 100,000). **d** Western blot of CD63 (30–60 kDa) under non-reducing conditions

The mammary epithelial cell line MCF10A, and the estrogen-receptor-positive (luminal subtype) MCF7 and triple-negative (basal subtype) MDA-MB-231 breast cancer cell lines were plated at 5 × 10^6^ in cell culture medium supplemented with exosome-depleted serum for 3 days. Whole exosomes were isolated from the conditioned medium, diluted in PBS and subjected to nanoparticle tracking analysis (Fig. 1a). While the mean sizes of MCF7 and MDA-MB-231 exosomes were comparable, the MCF10A exosomes were slightly larger on average. Next, we visualized the MCF7 and MDA-MB-231 isolated exosomes by electron microscopy and found exosomes of the typical size and morphology (Fig. 1b). The isolated exosomes had detectable CD63, an established marker for exosomes, identified by immunogold labeling (Fig. 1c) and by western blot analysis (Fig. 1d). These results indicate our capability of isolating and characterizing human breast cancer exosomes.

**Fig. 2:**
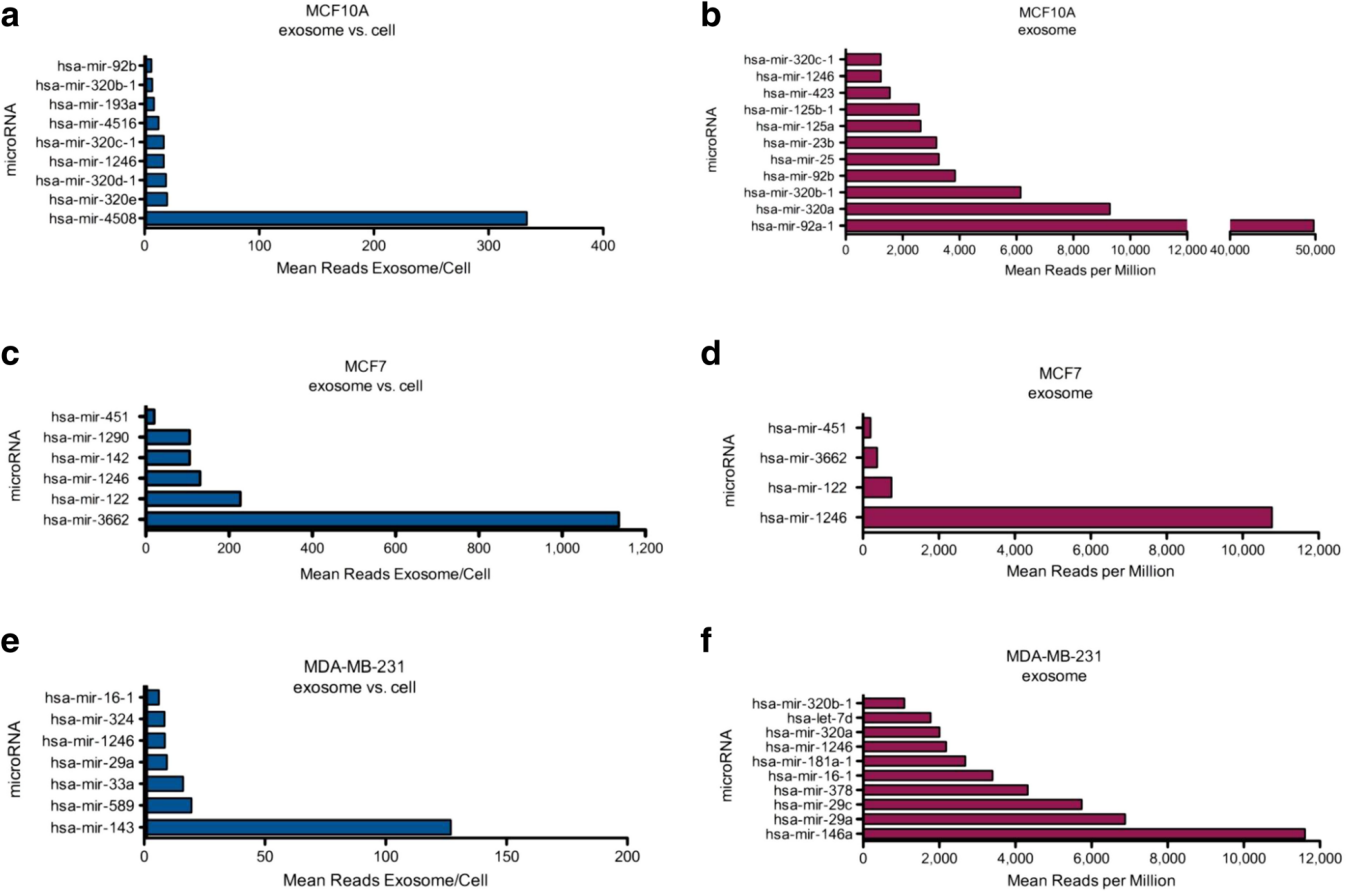
Small RNA next generation sequencing analysis of microRNAs in mammary epithelial cells and breast cancer cell lines versus exosomes. Cellular and exosome RNA was isolated from normal mammary cells (MCF10A) and breast cancer cell lines (MCF7 and MDA-MB-231). microRNAs were differentially expressed in MCF10A (**a**), MCF7 (**c**) and MDA-MB-231 (**e**) exosomes with a fold-change ≥6.0 (exosome vs. cell, *p* < 0.05) with mapped reads ≥ 200 are plotted. The abundance of the microRNAs highly expressed in exosomes vs. the cellular RNA in MCF10A (**b**), MCF7 (**d**) and MDA-MB-231 (**f**) are plotted by their mean number of reads per million reads

To determine whether the microRNA profiles of mammary epithelial cells and breast cancer cells may differ, total RNA was isolated from the cells and exosomes and a small RNA library was prepared using equal quantities of RNA. The resulting cDNA library was sequenced and the reads were mapped onto the human genome (complete RNA sequencing data is available in Additional file 1). We first compared the cellular microRNA content to the matched exosome microRNAs for each cell line (Fig. 2). In MCF10A, 168 microRNAs were differentially expressed in the exosomes compared to the cells, 33 of these microRNAs were higher in the exosomes than the cells (selectively encapsulated by exosomes), 9 of these were at least six-fold greater in the exosomes versus the cell (Fig. 2a), and 11 of the selectively secreted microRNAs were detected at a level of 1000 reads or more (Fig. 2b). In MCF7, 196 microRNAs were differentially expressed in the exosomes compared to the cells, 6 of them were at least 20-fold higher in the exosomes versus the cell (Fig. 2c), and 4 of the 6 were detected at a level of 200 reads or more (Fig. 2d). In MDA-MB-231, 101 microRNAs were differentially expressed in the exosomes compared to the cells, with 63 higher in the exosome versus the cell; 7 of these were at least six-fold greater in the exosome versus the cell (Fig. 2e) and 10 were detected at a level of 1000 reads or more (Fig. 2f). Interestingly, the top ranked microRNAs that are selectively encapsulated by exosomes differed among MCF7, MDA-MB-231, and MCF10A cells (Fig. 2 and Table 1), indicating different profiles of cancer exosome microRNAs compared to normal epithelial exosome microRNAs.

**Table 1:** microRNAs selectively secreted to exosomes from mammary epithelial cells and breast cancer cells

MCF10A			MCF7			MDA-MB-231		
microRNA	Fold-change	Mean reads	microRNA	Fold-change	Mean reads	microRNA	Fold-change	Mean reads
hsa-mir-4508	333.36	94.73	hsa-mir-3662	1136.26	375.59	hsa-mir-143	126.92	11.87
hsa-mir-320e	19.64	446.61	**hsa-mir-122**	227.25	751.19	hsa-mir-589	19.61	18.34
hsa-mir-320d-1	18.70	478.18	**hsa-mir-1246**	130.37	10773.97	hsa-mir-33a	16.15	15.10
hsa-mir-1246	16.73	1236.06	hsa-mir-142	105.21	34.78	hsa-mir-29a	9.46	44.22
hsa-mir-320c-1	16.67	1231.55	hsa-mir-1290	105.21	34.78	**hsa-mir-1246**	8.61	2181.03
hsa-mir-4516	12.17	103.76	**hsa-mir-451**	20.34	201.71	hsa-mir-324	8.46	23.73
hsa-mir-193a	8.33	94.73				hsa-mir-16-1	6.11	3398.83
hsa-mir-320b-1	6.70	6148.72						
hsa-mir-92b	6.07	3848.03						

The microRNAs with a fold-change ≥6.0 in the exosome vs. the cellular content and mapped reads ≥30 are shown. The fold-change was calculated by normalizing the reads to the number of mapped reads. microRNAs in bold were further evaluated in this study

Next, we asked whether any microRNAs are highly present in breast cancer exosomes compared to mammary epithelial exosomes. Our assumption was that microRNAs highly enriched in breast cancer exosomes over normal epithelial exosomes are potential circulating biomarkers for breast cancer. Pairwise analysis showed that 10 microRNAs were found at greater levels in MCF7 exosomes compared to MCF10A exosomes, whereas 132 microRNAs were found at greater levels in MDA-MB-231 exosomes compared to MCF10A exosomes. We listed the top 8–10 of these microRNAs in MCF7 and MDA-MB-231 cells, considering their absolute abundance in the exosomes (Table 2). As shown in Table 2, miR-1246 was most enriched, followed by miR-122 in MCF7 exosomes, and miR-21 was most enriched in MDA-MB-231 cells, followed by let-7a. These microRNA species, along with others shown in Table 2 were considered potential exosome microRNAs that may serve as biomarkers for breast cancer.

**Table 2:** microRNAs in breast cancer cell line exosomes compared to mammary epithelial cell exosomes

MCF7 vs. MCF10A exosome					MDA-MB-231 *vs.* MCF10A exosome				
microRNA	MCF10A mean reads	MCF7 mean reads	Fold-change	*P* value	microRNA	MCF10A mean reads	MDA-MB-231 mean reads	Fold-change	*P* value
**hsa-mir-1246**	**1236.06**	**10773.97**	**8.72**	**0.00E + 00**	**hsa-mir-21**	**9527.59**	**157280.08**	**16.51**	**1.00E-20**
**hsa-mir-122**	**18.04**	**751.19**	**41.63**	**0.00E + 00**	**hsa-let-7a-1**	**1470.64**	**30328.39**	**20.62**	**1.00E-20**
hsa-mir-3662	0.45	375.59	832.59	0.00E + 00	hsa-let-7f-1	1163.88	29759.95	25.57	1.00E-20
**hsa-mir-451**	**22.56**	**201.71**	**8.94**	**2.99E-10**	hsa-let-7e	744.34	12747.48	17.13	1.00E-20
hsa-mir-200a	0.45	86.94	192.73	9.02E-08	hsa-mir-30e	789.45	12061.46	15.28	1.00E-20
hsa-mir-203	4.51	45.21	10.02	2.27E-03	hsa-mir-146a	76.69	11607.35	151.35	1.00E-20
hsa-mir-142	0.45	34.78	77.09	7.23E-04	hsa-let-7i	2133.78	11003.31	5.16	1.00E-20
hsa-mir-1290	0.45	34.78	77.09	7.23E-04	hsa-let-7 g	387.96	8518.10	21.96	1.00E-20
					hsa-mir-29a	360.89	6881.79	19.07	1.00E-20
					hsa-mir-29c	288.71	5744.89	19.9	1.00E-20

The microRNAs with a fold-change ≥5.0 in each comparison and mapped reads ≥30 for MCF7 and ≥5000 for MDA-MB-231 are shown. The fold-change was calculated by normalizing the reads to the number of mapped reads. The *P* value was calculated using the likelihood ratio test. microRNAs in bold were further evaluated in this study

**Fig. 3:**
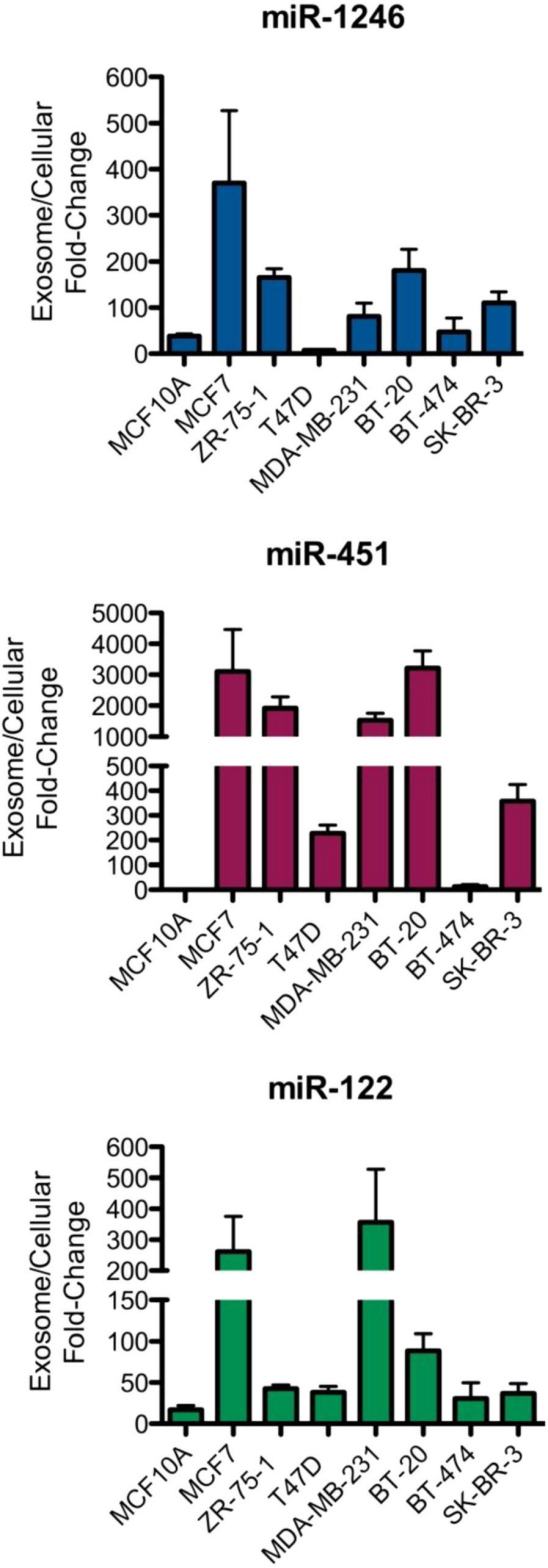
qRT-PCR analysis of microRNAs in breast cancer cell line exosomes versus mammary epithelial cell exosomes. qRT-PCR analysis of selected microRNAs that are abundantly present in MCF7, ZR-75-1, T47D, MDA-MB-231, BT-20, BT-474, and SK-BR-3 exosomes vs. MCF10A exosomes. Fold-change in expression is shown for the exosome microRNAs relative to their cellular microRNA levels and normalized to the spike-in cel-miR-54 control

We then selected several microRNAs that are selectively secreted or highly abundant in the breast cancer MCF7 exosomes (miR-1246, miR-451, and miR-122) for validation by real-time PCR analysis in seven breast cancer cell lines representing the various breast cancer subtypes (Fig. 3). The data indicated that these microRNA species are selectively secreted and enriched in breast cancer exosomes relative to their cellular content and compared to mammary epithelial exosomes, with a few exceptions (miR-1246 in T47D and miR-451 in BT-474). Based on their fold-change, the enrichment of all three microRNAs seemed to be more pronounced in the estrogen receptor (ER)+, progesterone receptor (PR)+ cell line-derived exosomes (MCF7, ZR-75-1), and the ER-, PR-, HER2- cell-line-derived exosomes (MDA-MB-213 and BT-20). These observations are consistent with the RNA sequencing results.

### miR-1246 is detected at significantly higher levels in the plasma exosomes of patient-derived orthotopic xenograft (PDX) mice compared to control mice

**Fig. 4:**
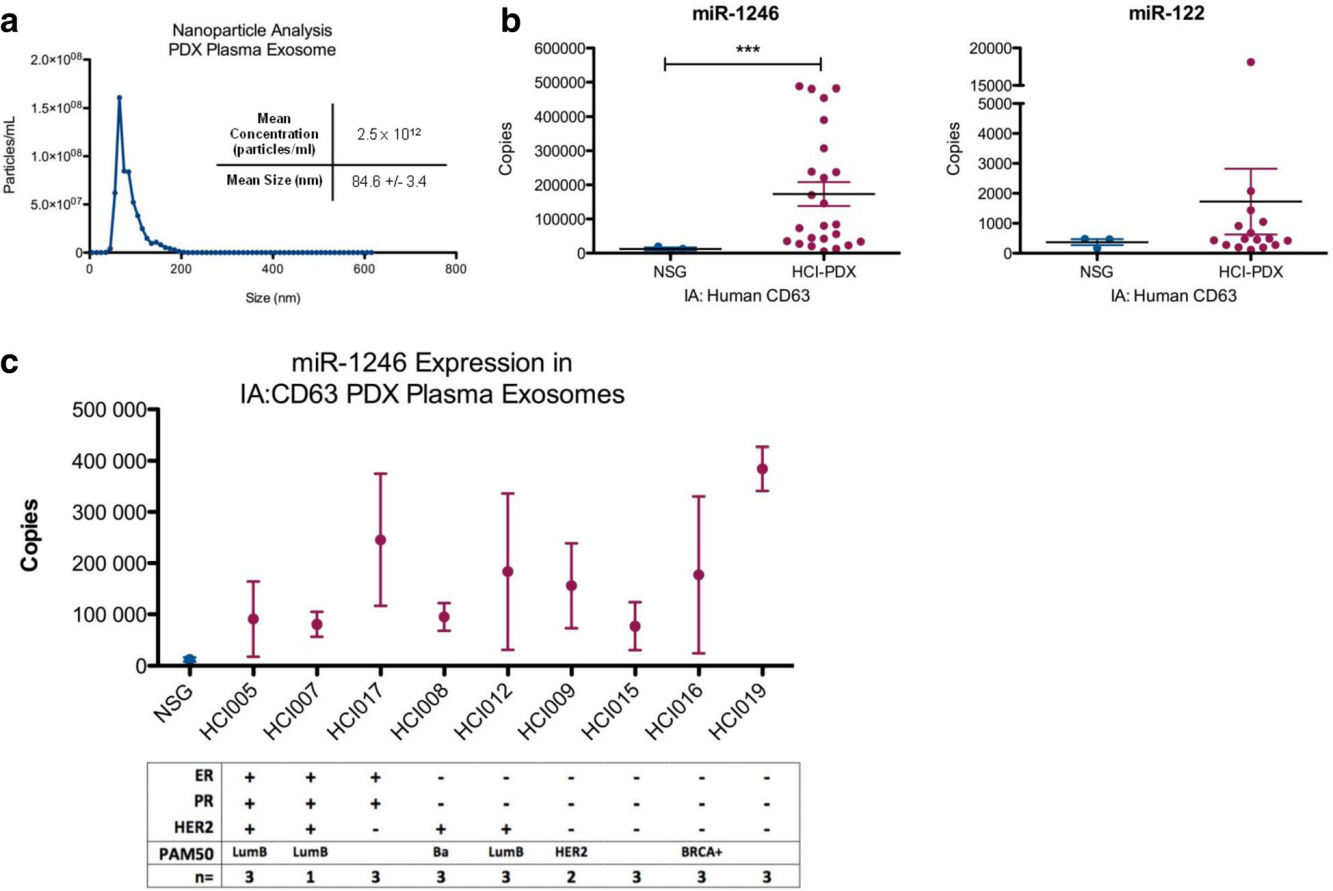
Characterization and microRNA expression analysis of human exosomes isolated from the plasma of patient-derived xenograft (*PDX*) mice. Exosomes were isolated from the plasma of Huntsman Cancer Institute human breast cancer orthotopic xenograft (HCI-PDX) and nod scid gamma (NSG) mice. **a** Exosomes were characterized by nanoparticle tracking analysis of 500X diluted exosomes (*n* = 3). **b**-**c** Exosomes were isolated from the mouse plasma exosome sample by magnetic-bead based immunoaffinity isolation using an antibody against human CD63, the total RNA was extracted and the expression of miR-1246 and miR-122 were evaluated by qRT-PCR analysis (absolute quantitation) in the immunoaffinity isolated human CD63-positive exosomes from the plasma of three NSG (*n* = 3, in triplicate) and nine HCI-PDX models (with available biological replicates as indicated in **c**, in triplicate); Student’s *t* test; ***p < 0.001. The corresponding clinical biomarkers including the estrogen receptor (*ER*), progesterone receptor (*PR*), and human epidermal growth factor 2 (*HER2*) amplification status and the PAM50 subtype of the human tumor (if known) are indicated and are available in Additional file 2

We reasoned that mice bearing human breast tumors should have circulating human exosomes and sought to determine whether selectively secreted human exosome microRNAs are detectable in the plasma of these mice. Plasma was collected from nine Huntsman Cancer Institute-PDX (HCI-PDX) mice models [20] and three non-tumor-bearing nod-scid gamma (NSG) mice at the time of killing (see PDX information in Additional file 2). Whole exosomes were isolated from the plasma of a PDX mouse and analyzed by nanoparticle tracking analysis (Fig. 4a). The exosomes were further purified by magnetic-bead-based immunoaffinity isolation using an antibody against human CD63. Total RNA was extracted from the bead-bound exosomes and the expression of miR-1246, miR-451 and miR-122 were measured by qRT-PCR.

As shown in Fig. 4b, both miR-122 and miR-1246 were detected in the human-CD63-positive exosome population from the PDX mice, with miR-1246 levels being significantly higher (*p* < 0.001) in PDX mouse plasma compared to the NSG control plasma. miR-451 was not detectable in either the PDX mouse plasma or the NSG plasma. While the expression of plasma exosome miR-1246 was higher in mice with any subtypes of breast cancer, those mice with ER- and PR- tumors had the highest levels (Fig. 4c). These data suggest that breast cancer exosomes are released to the circulation in human breast cancer PDX mouse models and that human-exosome-specific microRNAs can be detected in the mouse plasma.

### miR-1246 and miR-21 are highly expressed in plasma exosomes from patients with breast cancer

**Fig. 5:**
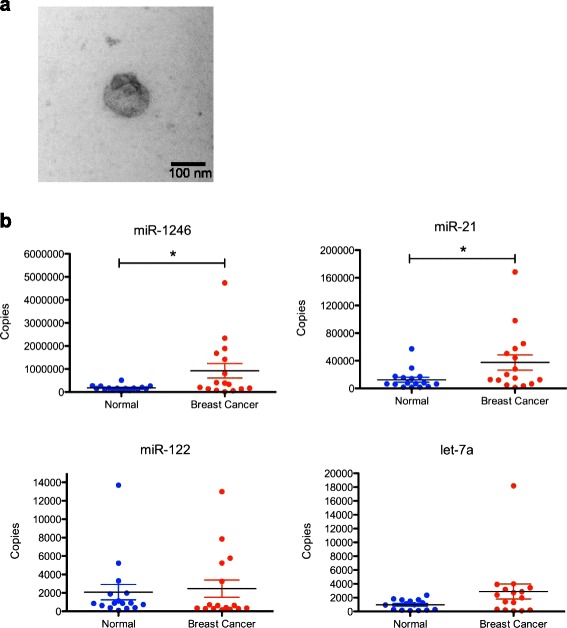
qRT-PCR analysis of exosome microRNA expression in normal plasma and plasma from patients with breast cancer. Plasma samples were collected under an Institutional Review Board approved protocol from healthy women with no history of cancer (*n* = 16, mean age 42 years, age range 27–66) and women with breast cancer (*n* = 16, mean age 59.6 years, range 42–80; see Table 3). Exosomes were isolated from plasma samples using the Exoquick reagent and total RNA was extracted. **a** Human plasma exosomes were visualized by electron microscopy (×30,000). **b** Candidate microRNA expression was measured in normal plasma (*n* = 16) and plasma from patients with breast cancer (*n* = 16) by qRT-PCR. Student’s *t* test, *p < 0.05

To determine whether enriched exosome microRNAs could be detected in circulating exosomes from breast cancer patients we collected 16 plasma samples from women with no history of breast cancer and 16 plasma samples from women with breast cancer (mostly ER+, grade 2–3; see Table 3 for patient sample information). Plasma exosomes were isolated and verified by electron microscopy (Fig. 5a). Total RNA was isolated from the whole exosome population and the expression of several selectively secreted microRNAs (cell to the exosome) and/or abundantly expressed (cancer exosomes versus the MCF10A exosomes) (miR-1246, miR-21, miR-122, and let-7a) was analyzed by real-time PCR. As shown in Fig. 5, the expression of plasma exosome miR-1246 (*p* = 0.03) and miR-21 (*p* = 0.04) was significantly higher in the group of patients with breast cancer as compared to the normal population (Fig. 5b). There was no significant correlation with patient clinicopathological characteristics such as tumor stage, grade, or size, likely due to the limited sample size (Additional file 3).

**Table 3:** Clinicopathological features of patients with breast cancer included in the study

Plasma sample	Age	ER status	PR status	HER2 status	Grade	Stage^a^
PL7	48	-	+	-	3	NA
PL8	67	+	+	-	2	IIA
PL9	56	+	-	-	2	NA
PL10	56	+	+	-	3	IIB
PL11	63	+	+	-	1	IA
PL12	53	+	+	-	3	NA
PL13	51	NA	NA	NA	3	IIIA
PL14	52	-	-	+		0
PL15	71	+	+	-	2/1	IA
PL16	80	+	+	-	2	IIA
PL17	75	+	-	+		NA
PL18	51	+	+	-	1	IIA
PL19	68	+	+	-	3	IIB
PL20	56	+	+	+	2	NA
PL21	65	+	+	-	2	NA
PL22	42	+	+	-	2	IIA
Mean age	59.6					
Median age	56.0					

^a^Pathologic TNM tumor staging, (American Joint Committee on Cancer Care 7^th^ Edition). *ER* estrogen receptor, *PR* progesterone receptor, *HER2* human epidermal growth factor receptor 2 amplification, *NA* not available

**Fig. 6:**
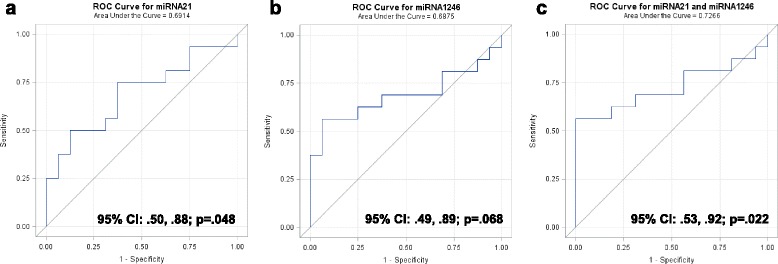
Receiver operator characteristic (*ROC*) analysis of microRNA expression in plasma exomes from normal subjects and patients with breast cancer. ROC curves for classifying plasma exosomes (breast cancer vs. normal) were produced using each microRNA expression value for miR-21 (**a**) and miR-1246 (**b**) separately, and for miR-21 and miR-1246 combined (**c**). For each patient sample and each microRNA, the average of three replicate expression values was computed. The resulting microRNA expression values were standardized for the statistical analysis. The area under the curve (AUC) with 95 % CI were computed for each ROC curve. The Wilcoxon-Mann-Whitney test was used to test the null hypothesis that the AUC is equal to 0.5 (i.e., no predictive power)

ROC curves were constructed separately for miR-21 (Fig. 6a) and miR-1246 (Fig. 6b), and combined (Fig. 6c) to compare the diagnostic value of miR-1246 and miR-21 to predict breast cancer. The AUC for miR-21 was 0.69 (95 % CI 0.50, 0.88; *p* = 0.048) and for miR-1246 was 0.69 (95 % CI 0.49, 0.89; *p* = 0.068), indicating fair predictive power. When miR-1246 and miR-21 were combined, the AUC increased to 0.73 (95 % CI 0.53, 0.92; *p* = 0.022). These data indicate that breast cancer exosome microRNAs can be detected in the circulation in patients with breast cancer, and that their expression could differentiate patients with breast cancer from healthy control subjects.

## Discussion

The most interesting finding of the present study was that breast cancer exosome microRNAs possess unique signatures of the malignancy, and could serve as circulating biomarkers for breast cancer. Circulating microRNAs have been proposed as biomarkers that may aid in cancer diagnosis and prognosis, and for treatment monitoring. Indeed, many studies have evaluated this possibility, yet very few have reported consistent results [21]. Our current understanding of circulating microRNAs is that there are distinct populations of microRNAs either within membrane-bound vesicles or associated with protein complexes, which may likely originate from different cell types and reflect different release mechanisms [22]. This suggests that plasma exosome purification strategies may serve to enrich those microRNAs that originate from certain cell types.

In this study, we focused our efforts on cancer exosomes as specific carriers of microRNAs in order to more specifically analyze circulating cancer microRNAs. This is in contrast to many published biomarker studies in the field of breast cancer research, where efforts are usually focused solely on profiling circulating microRNAs in patients with breast cancer [23, 24]. We began this study by characterizing breast cancer exosome microRNA signatures using cell line-derived exosomes. Genome-wide profiling of the microRNA content of breast cancer cell line exosomes revealed that certain microRNAs are selectively enriched in cancer exosomes vs. exosomes from normal epithelial cells. This phenomenon provides us with a rich resource for biomarker discovery. PDX mouse models were then applied to verify our findings from cell lines, and one of the microRNAs identified in cell line studies, miR-1246, was abundant, and its level was significantly higher in the plasma exosomes from the PDX mice compared to the control mice. This observation supports the concept that breast cancer exosome microRNAs are released from the primary tumor site and are detectable in the circulation.

We then extended our efforts to patient plasma samples to determine whether the candidate exosome microRNAs are associated with the presence of a breast tumor. Based on the selective enrichment and absolute abundance, we identified miR-1246, miR-122, miR-21, and let-7a as candidate exosome microRNAs that may serve as biomarkers indicative of breast cancer. The confirmation that miR-1246 and miR-21 levels are significantly higher in plasma exosomes from patients with breast cancer vs. those from normal control subjects indicates that circulating breast cancer exosome microRNAs are promising biomarkers for this malignancy.

The fairly consistent results from cell line exosome characterization to plasma exosome microRNA detection in PDX mice and breast cancer patients are in agreement with a recent massive genomic and proteomic study indicating that breast cancer cell lines are reasonable models for analyzing context-dependent gene expression, including microRNA expression [25]. While direct profiling of circulating exosome microRNAs has been a common approach for cancer biomarker identification, the challenge to this approach is that the exosomes found in the circulation are a heterogeneous mixture of exosomes from various other cells and tissues, which compromises the specificity of the identified microRNA signatures. Our results indicate that deriving information from a cell line study and confirming the observation through investigations in mice and humans seems to be a valid alternative approach for development of circulating biomarkers.

Our finding that the levels of the selectively secreted and highly enriched exosome miR-1246 were significantly higher in the plasma of PDX mice vs. the NSG control mice indicates that the PDX mouse model is suitable for a breast cancer biomarker study. The PDX mouse model has recently been extensively used for breast cancer research due to its suitability in recapitulating the heterogeneity and behavior of the original tumor. However, most studies with PDX mice have been focused on verifying breast cancer biology and screening of therapeutic agents to provide evidence to facilitate personalized medicine [26, 27]. To our knowledge, the PDX mouse model has never been applied for investigating plasma exosome microRNAs as biomarkers for breast cancer. Considering that breast cancer PDX mouse models are well-established [20], the use of these mouse models for a biomarker study is rather appealing. The advantages of using a PDX mouse model to evaluate circulating exosome microRNAs as biomarkers for breast cancer may include first, the use of a cross-species model (human and mouse) that renders an opportunity to specifically enrich human cancer exosomes from the mouse plasma and more specific detection of cancer exosome microRNAs in the circulation; second, the variety of tumors that may be grown, including well-established subtypes of human breast cancer in PDX mice, provides a unique platform to evaluate cancer exosome microRNAs across subtypes; and last, the possibility to analyze circulating human exosome microRNAs at various stages of tumor growth and progression, which could help to determine whether plasma exosome microRNAs can serve as biomarkers for early detection. Further exploration of the PDX mouse model for the study of cancer biomarkers is warranted.

We have detected plasma exosome miR-21 and miR-1246 at significantly higher levels in patients with breast cancer vs. healthy women. Based on previous reports, both miR-21 and miR-1246 are cancer-associated microRNA species. miR-21 is considered an oncogenic microRNA that is known to be overexpressed in both male and female invasive breast cancer relative to normal breast tissue [4, 5, 28]. This over-expression positively correlates to the size, stage, grade and proliferation rate of the tumor, and is associated with metastatic breast cancer [29, 30]. Differential expression of serum miR-21 has been previously identified in circulating exosomes from patients with lung cancer [31], melanoma [32], and breast cancer [33] with limited diagnostic value.

Compared to miR-21, miR-1246 is relatively less investigated in cancer but was previously identified as selectively released from breast cancer cells [14], and has been found at greater levels in serum from patients with breast cancer compared to their matched tumor tissues [34]. Furthermore, a most recent study indicated that serum miR-1246 is elevated among patients with invasive breast cancer [35]; these observations are consistent with our current findings. In addition, miR-1246 has also been found to be elevated in serum from patients with esophageal cancer [36], colon cancer [37], and pancreatic cancer [38]. Thus, our observation that the levels of plasma exosome miR-21 and miR-1246 are significantly higher in patients with breast cancer is in line with previous reports and suggests that these two exosome microRNA species are selectively enriched and significantly elevated in plasma exosomes potentially among a spectrum of different types of human cancer. While these microRNAs may not themselves be strong predictors of breast cancer alone, they may serve as a companion tools following other screening procedures such as individual breast cancer risk assessment and mammography.

It is important to note that recent studies have evaluated exosome microRNA expression and their association with cancer, including those of the prostate [39], lung [40], ovary [11], liver [41], colorectal [42], skin [32], pancreas [38], and glioblastoma [43]. Furthermore, a recent study evaluated the levels of previously identified circulating microRNAs (miR-373, miR-101 and miR-372) in cell-free versus extracellular vesicle preparations from the serum of patients with breast cancer [44]. However, to date, no study has fully characterized the exosome microRNA content of breast cancer cells and examined the levels of microRNAs in circulating exosomes from the plasma of human-tumor bearing mice and patients with breast cancer as biomarkers for breast cancer, thus making this study novel and unique.

Given the magnitude of breast cancer incidence and the lack of sensitive and specific biomarkers for early detection of this malignancy [2, 3], the results from this study support further investigations of the potential of exosome microRNAs as effective biomarkers for breast cancer. Whereas most of the studies mentioned above, including the current study, identified exosome microRNA species that are significantly elevated in the circulation in patients with cancer, the sensitivity and specificity of these exosome microRNAs are not yet satisfactory. This indicates that plasma exosome populations are heterogeneous and may be derived from all types of cells, especially blood cells. In this context, while current circulating exosome microRNA detection may not be superior to circulating microRNA analysis, in terms of the sensitivity and specificity for detecting cancer, it does point out an important new direction by which specific and sensitive plasma biomarkers can be developed. Therefore, methods to selectively isolate cancer exosomes from the circulation becomes a key step in the future development of exosome biomarkers for breast cancer.

## Conclusion

In conclusion, we have demonstrated that certain microRNA species, such as miR-21 and miR-1246, are selectively enriched in human breast cancer exosomes, detectable in plasma from breast cancer PDX mice, and significantly elevated in plasma from patients with breast cancer. Our results support the notion that circulating exosome microRNA profiles can serve as an important companion diagnostic tool for breast cancer. Further studies are required to specifically isolate cancer exosomes from patient plasma and establish exosome microRNA signatures indicative of breast cancer.

## Additional files

Additional file 1: Spreadsheet containing the complete microRNA sequencing data for cell versus exosomes and normal vs. cancer exosomes pairwise comparisons. (XLSX 109 kb) Additional file 2: Table containing the clinicopathological features of the patient-derived xenograft (PDX) mice used in this study. (DOCX 13 kb) Additional file 3: Table containing the correlation analysis of the clinicopatholigical features and microRNA expression in patient plasma exosomes. (DOCX 43 kb)

## Abbreviations

bp: Base pair(s)
cDNA: complementary DNA
DMEM: Dulbecco’s modified Eagle’s medium
ER: Estrogen receptor
FBS: Fetal bovine serum
HER2: Human epidermal growth factor receptor 2
IBC: Invasive breast cancer
kDa: kiloDaltons
NSG: Nod scid gamma
PBS: Phosphate-buffered saline
PDX: Patient-derived xenograft
PR: Progesterone receptor
ROC: Receiver operator characteristic
RPMI: Roswell Park Memorial Institute
