# Supplementary material for: Plasma exosome microRNAs are indicative of breast cancer
Source: Breast Cancer Res. 2016 Sep 8;18(1):90. doi: 10.1186/s13058-016-0753-x (PMC5016889; doi:10.1186/s13058-016-0753-x)
Supplement: Additional file 2: — Table containing the clinicopathological features of the patient-derived xenograft (PDX) mice used in this study. (DOCX 13 kb) [file 13058_2016_753_MOESM2_ESM.docx]

| **ID** | **Source** | **Primary Diagnosis** | **ER Status** | **PR Status** | **HER2 Status** | **PAM 50** | **Reference** |
| --- | --- | --- | --- | --- | --- | --- | --- |
| HCI-005 | Pleural Effusion | Mixed IDC & ILC, Stage 2B | + | + | + | LumB | DeRose 2011  (Ref 21) |
| HCI-007 | Pleural Effucion-tap 4 | Mixed IDC & ILC, Stage 2B | + | + | + | LumB | DeRose 2011  (Ref 21) |
| HCI-008 | Pleural Effusion | Inflammatory breast cancer | - | - | + | Basal-like | DeRose 2011  (Ref 21) |
| HCI-009 | Ascites | IDC | - | - | - | HER2-like | DeRose 2011  (Ref 21) |
| HCI-012 | Pleural Effusion | IDC | - | - | + | LumB | DeRose 2011  (Ref 21) |
| HCI-015 | Brain Mets | IDC | - | - | - | na | unpublished |
| HCI-016 | 1° breast tumor | IDC; BRCA+ | - | - | - | na | unpublished |
| HCI-017 | 1° biopsy of breast tumor | IDC | + | + | - | na | unpublished |
| HCI-019 | 1° breast tumor | IDC | - | - | - | na | unpublished |
| Abbreviations: IDC, invasive ductal carcinoma; BRCA, breast cancer gene; na, not available. | | | | | | | |

**Additional file 2**

Clinical and pathological features of the patient-derived xenograft (PDX) mice used in this study.
