# Supplementary material for: Plasma exosome microRNAs are indicative of breast cancer
Source: Breast Cancer Res. 2016 Sep 8;18(1):90. doi: 10.1186/s13058-016-0753-x (PMC5016889; doi:10.1186/s13058-016-0753-x)
Supplement: Additional file 3: — Table containing the correlation analysis of the clinicopatholigical features and microRNA expression in patient plasma exosomes. (DOCX 43 kb) [file 13058_2016_753_MOESM3_ESM.docx]

| **miR-1246** | | | | **miR-21** | | |
| --- | --- | --- | --- | --- | --- | --- |
|  | Correlation | | P value |  | Correlation | P value |
| Grade | 0.1664 | | 0.5697 | Grade | -0.05547 | 0.8443 |
| Stage | 0.2988 | | ns | Stage | 0.08240 | ns |
| Tumor Size | -0.02395 | | ns | Tumor Size | 0.05085 | ns |
| ns=non-significant | |  | | | | |

**Additional Data File 2.** Correlation analysis of clinicopatholigical features and microRNA expression in patient plasma exosomes.
